# Supplementary material for: Chromothripsis during telomere crisis is independent of NHEJ, and consistent with a replicative origin
Source: Genome Res. 2019 May;29(5):737–49. doi: 10.1101/gr.240705.118 (PMC6499312; doi:10.1101/gr.240705.118)
Supplement: Supplemental Material [file supp_gr.240705.118_Supplemental_file_1.zip › contigs/annotated_contigs/DB51/contig.3.DB51_length_688_mean_cov_6.75872093023.docx]

**DB51_length_688_mean_cov_6.75872093023**

```
TACCCTCTTAGGTTCTGCAGCTGGGCCTGAGAATTAAACTGACAAAAGACAGAGAAACAGGAGAAAAGCATAGACATTTTATTTGATGT
 >chr6:14465561-14465778 - E=9e-119 p=2e-02
TAATATTTTTGCATGGCATAGGAGACTTCACAGAAAAAAGAAATGAAAAGGCCCGAGAGGGTATATACCATTTCAACAAAGAATGATCA

ACTGTGGAGATGTGAAAGATCAACTGTGGAGCGAAAGG|A|CTTAGATTTTATTTATTTATTTAGAGACGAAGTCTCCCTCTGTCACC|
                                       >chr6:10510302-10510354 - E=2e-19
CAGG|ACAACTGTGATTGCCTGAAGTTGAAGAAATATAGTGTCCCTTGCATTCAAAGGTGACATCAGTGATGGTGATGGTGGCTCTGCA
>chr6:10513785-10514209 + E=2e-240
TGGGAAAAAGAGGTGCCAAATGAACACACATTCCACCTGTACTCCACATGGGAAAAAAACCCTGAGCTGGCAGCTGTGGCCGATTTGCT

AGGGTCTTAATTTGTGGTTGACCTAGACAGAGGTGCCCAACTGTGCCTGGCTTTTGTGAAGTGGTCAGCTGGCCCCACTGGCTCATGTT

CAAAGAGTCACCTGATTTTTTAAATAGAAGAGCTACCGACTTGTGGTTGATCCTCCTTCTTTTAAGTAAATGGTCATATTGCACTTCTT

GCTGCAAATAAGTTGCTAGAGCACATTTCTCAAGTCTGGGCAGGGACCTGCAGACTGACTTCAGCAATG
```
